# Wilson Disease and the COVID-19 pandemic: exploring patients’ mental health and vaccination attitudes in a longitudinal study

**DOI:** 10.3389/fpsyg.2024.1326802

**Published:** 2024-05-13

**Authors:** Ayse K. Coskun, Adem Aydin, Sumeyra Tosun, Uyen To, Susan Rubman, Michael L. Schilsky, Paula C. Zimbrean

**Affiliations:** ^1^Department of Surgery, Yale University School of Medicine, New Haven, CT, United States; ^2^Medgar Evers College, CUNY, Brooklyn, NY, United States; ^3^Medicine and Surgery, Yale University School of Medicine, New Haven, CT, United States; ^4^Department of Psychiatry, Yale University School of Medicine, New Haven, CT, United States

**Keywords:** Wilson Disease, COVID-19, mental health, vaccination, cognitive functions

## Abstract

**Introduction:**

The COVID-19 pandemic significantly impacted the mental health of individuals with chronic conditions such as Wilson’s Disease (WD). This study investigates stress, anxiety, depression, quality of life, cognitive function, vaccination rates, infection rates, and perceptions related to the pandemic and vaccines among WD patients.

**Methods:**

The study analyzed COVID-19 perceptions and vaccine attitudes of 62 adult WD patients enrolled in the international multisite WD Registry. A subgroup of 33 participants completed a series of mental health scales. The effect of working essentially, income loss, wellness activity initiation, and infection of COVID-19 during the pandemic was observed.

**Results:**

Results indicate that, overall, the pandemic did not exacerbate anxiety or cognitive function in WD patients but did lead to increased depression among essential workers. Patients experiencing income loss exhibited higher levels of stress and anxiety. Despite these challenges, WD patients showed high vaccination rates and positive attitudes towards vaccines.

**Discussion:**

The findings underscore the significant impact of the pandemic on the mental health of WD patients.

## Introduction

Wilson’s disease (WD), previously known as hepatolenticular degeneration, is a rare genetic disorder characterized by defective copper transport and metabolism, leading to copper accumulation in various tissues, including the liver, brain, cornea, and kidneys. The disease is associated with a spectrum of psychological difficulties. Patients with WD often experience neuropsychiatric symptoms, significantly impacting their mental health and well-being, with an estimated prevalence of 30–40%. However, the exact prevalence of psychiatric disorders in WD remains uncertain due to limited prospective cohort studies. Given the susceptibility of this population to psychiatric issues (Zimbrean and Schilsky, 2014), especially during the COVID-19 pandemic, it is crucial to assess the impact on mental health and relevant factors. This study aims to examine the impact of COVID-19 and its associated consequences on patients with chronic liver diseases, including WD.

During the early stages of the pandemic, the significant impact of COVID-19 on mental health became apparent. SARS-CoV-2 is found to have neurotropic effects that can lead to various neurological symptoms by affecting the central nervous system or triggering immune responses and inflammation, contributing to increased neuropsychiatric symptoms in survivors (e.g., Troyer et al., 2020; Vindegaard and Benros, 2020). Stress and uncertainty related to the virus, along with mandatory isolation from educational, social, and work activities due to widespread lockdowns, have led to changes in mental health needs (Moreno et al., 2020). Compared to pre-pandemic conditions, the general population has experienced a decline in psychological well-being, with increased levels of anxiety, stress, and depression (Wang et al., 2020). Patients with chronic conditions, including WD, have shown increased susceptibility to morbidity and mortality from SARS-CoV-2 infection, leading to heightened levels of anxiety, stress, and depression (Lee et al., 2020; Pedrozo-Pupo and Campo-Arias, 2020).

During the COVID-19 pandemic, guidelines were issued to assist healthcare providers in delivering optimal care for individuals with chronic liver disease, with a particular focus on understanding the effects of the virus on liver function and how pre-existing liver conditions might influence disease progression (Boettler et al., 2020). Despite these efforts, the impact of the pandemic on the mental health of liver disease patients, including various subgroups within this population, remains to be fully explored.

As stated earlier, individuals with Wilson disease face a range of psychological challenges. One of the prominent psychological challenges experienced by Wilson’s disease patients is mood disorders. Research has shown a high prevalence of mood disturbances, with depression being a common manifestation (Zimbrean and Schilsky, 2014; Medici et al., 2018). These mood disorders can exacerbate the overall psychological burden experienced by individuals with Wilson’s disease. Cognitive impairment is another critical aspect of psychological difficulties in this population. Memory deficits and executive dysfunction are prevalent cognitive symptoms among Wilson’s disease patients (Zimbrean and Schilsky, 2014; Medici et al., 2018). These cognitive impairments can impact daily functioning and quality of life. Furthermore, psychosis is observed in some cases, adding to the complexity of psychological difficulties associated with Wilson’s disease. Psychotic symptoms may include hallucinations and delusions (Zimbrean and Schilsky, 2014; Medici et al., 2018). Anxiety disorders, such as generalized anxiety disorder and panic disorder, can also occur (Zimbrean and Schilsky, 2014). These anxiety-related problems can contribute to the overall psychological distress of affected individuals. Moreover, personality changes have been documented in Wilson’s disease patients. These changes can manifest as alterations in behavior, interpersonal relationships, and emotional regulation. In conclusion, Wilson’s disease is linked to a range of psychological difficulties, encompassing mood disorders, cognitive impairment, psychosis, anxiety disorders, and personality changes. Understanding and addressing these psychological challenges are crucial components of the holistic care and management of individuals affected by this condition during the pandemic.

Individuals have been significantly impacted by the changes brought about by COVID-19. Essential personnel, including those in healthcare, law enforcement, food and agriculture, and energy industries faced a heightened risk of contracting the virus due to their on-site work requirements. In contrast, non-essential personnel have had to adapt to new work-from-home arrangements which posed challenges to their productivity (Chong et al., 2020; Trougakos et al., 2020). Some employees have also experienced job loss or a reduction in their income sources. Global attempts to alleviate the socioeconomic and health-related consequences of the pandemic initially relied on preventative measures (Calina et al., 2020) such as wearing masks, vaccinating, and social distancing. Given the psychological vulnerability of WD patients, it is essential to analyze their perspectives on crucial aspects such as COVID-19 vaccination, work arrangements, and income loss. Understanding these factors will aid in identifying potential issues and developing appropriate interventions.

In summary, this study seeks to explore WD patients’ understanding, attitudes, and perceptions concerning the COVID-19 pandemic and vaccination efforts. Additionally, it aims to assess the pandemic’s impact on the mental well-being of WD patients, focusing on changes in self-reported depression, anxiety, stress levels, quality of life, cognitive function, and associated factors. The specific objectives include evaluating: (i) the vaccination rate and preference (ii) patients’ experiences during the pandemic (iii) the infection rate (iv) the perception of the pandemic among the WD population (v) the course of stress, anxiety, and depression before and during the pandemic (vi) the effect of the pandemic on quality of life (vii) changes in cognitive function and (viii) any clinical, behavioral, or social factors that may have influenced the observed outcomes.

## Method

### Participants

**Figure 1:**
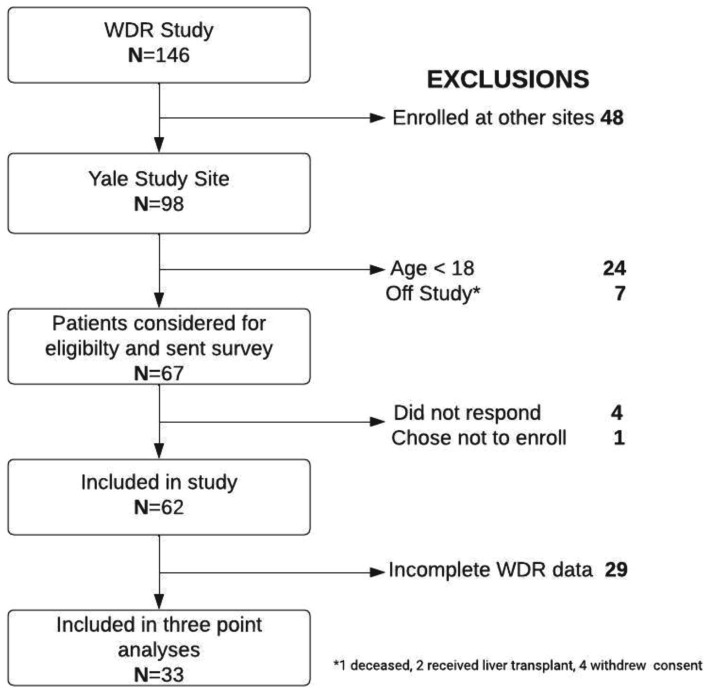
Flowchart of the population selection for the study, including patient recruitment, exclusion criteria, and refusals.

The multi-site Wilson’s Disease Registry (WDR) was established at Yale University to investigate the disease’s natural progression and to create and validate new diagnostic toolsets and biomarkers for diagnosis and treatment monitoring. The WDR Study enrolled consented subjects with confirmed diagnoses of WD, excluding transplant recipients, starting in December 2017. A total of 67 patients aged 18 or older enrolled in the WDR Study at Yale New Haven Hospital site. The survey was available to participants between November 23, 2022, and December 18, 2022. The return rate was 94%. Among the 67 patients who received the survey, 62 consented, 1 declined participation, and 4 did not respond. Among the 67 patients enrolled, we analyzed the 33^1^ participants who had mental health assessments at least twice before the pandemic and at least once during the pandemic (Figure 1). Additionally, the variation in sample sizes across different analyses occurred due to one participant who did not complete the MoCA test but completed the remaining mental health scales. In an effort to retain all participants in the study, we proceeded to analyze the available data for each participant. Consequently, when reporting the results of the MoCA (cognitive screening), we encountered a shortfall of one participant in the dataset (*N* = 32).

The study included 62 WDR patients, with 24 females (see Table 1). The ethnic composition of the cohort was predominantly white (87%), and the remaining (13%) participants consisted of Hispanics, African Americans, and Asians. The majority were married or had a partner (66%); others were single. The sample was highly educated, with most participants holding at least a college degree (AA: 3%, BA/BS: 45%, MA/MS: 29%, Ph.D.: 3%, Professional: 5%). The income levels varied among the participants.

**Table 1:** The summary of demographic features of the sample.

		*N* (62)	%
Gender	Female	24	38.71%
	Male	38	62.29%
Race	White	54	87.10%
	Hispanic	2	3.20%
	Black	1	1.60%
	Asian	4	6.50%
	Mix	1	1.60%
Marital status	Single	21	33.90%
	Married/partner	41	66.10%
Education	High school	4	6.50%
	Some college courses	5	8.10%
	AA	2	3.20%
	BA/BS	28	45.20%
	MA/MS	18	29.00%
	PhD	2	3.20%
	Professional	3	4.80%
Employment	Employed	42	67.70%
	Unemployed	4	6.50%
	Student	4	6.50%
	Retired	7	11.30%
	Disabled	2	3.20%
	Other	3	4.80%
Annual income	$0 to $19,999	3	4.80%
	$20,000-49,999	3	4.80%
	$50,000-89,999	13	21.00%
	$90,000-129,000	6	9.70%
	$130,000–149,999	2	3.20%
	$150,000+	23	37.10%
	Prefer not to say	12	19.40%

### Materials and measures

A vast number of mental health scales were employed to screen the participants’ mental health states. The scales were listed as follows:

#### Patient health questionnaire (PHQ)-9

It is a 9-item self-report inventory with items that assess the occurrence of DSM V criteria for Major Depressive Disorder (McDowell, 2006), using a Likert-type scale with responses ranging from 0 (not at all) to 3 (occurs nearly every day). It is designed to screen, diagnose, and monitor the severity of depression symptoms and has been validated in primary care (Kroenke et al., 2001; Beard et al., 2016) and behavioral health settings. Scores can range from 0 to 27. Typically, a total score of 10 or higher indicates the possible presence of depression. Its reliability score (Cronbach’s α) was found 0.89 and the criterion validity score was 88% (Kroenke et al., 2001).

#### Generalized anxiety disorder scale (GAD-7)

This scale (Spitzer et al., 2006) is a concise, self-report questionnaire employed to evaluate and measure the presence and severity of generalized anxiety disorder symptoms in individuals for clinical and research purposes. A total score of seven items ranges from 0 to 21, with greater scores indicating the severity of anxiety. Its reliability score (Cronbach’s α) was found 0.92 and the criterion validity score was 89% (Spitzer et al., 2006).

#### Perceived stress scale (PSS)

This scale (Cohen et al., 1983) is a psychological instrument that assesses an individual’s experience of stress by determining the degree to which life situations are considered stressful. Its reliability score (Cronbach’s α) was found 0.85. Individual scores on the PSS can range from 0 to 40 with higher scores indicating higher perceived stress. The PSS has shown strong correlations with other measures of stress and psychological distress, supporting its concurrent validity (Cohen et al., 1983).

#### The 12-item short form survey (SF-12)

This questionnaire (Ware et al., 1996) is a concise health questionnaire used to assess an individual’s overall health-related quality of life by measuring physical and mental health aspects through 12 items. Ware et al. (1996) reported Cronbach’s alpha values ranging from 0.70 to 0.89 for the different scales within the SF-12. Further, the SF-12 has shown criterion validity by accurately predicting various health-related outcomes, such as healthcare utilization, mortality, and clinical diagnoses. Each scale (physical and mental health) score ranges from 0 to 100, with higher scores indicating a better health condition.

#### MOCA: Montreal cognitive assessment (MoCA)

This scale serves as a comprehensive evaluation tool for multiple cognitive domains including memory, language, executive functions, visuospatial skills, calculation, abstraction, attention, concentration, and orientation (Nasreddine et al., 2005). MoCA scores range between 0 and 30. A score of 26 or over is considered to be normal. MoCA has demonstrated good test–retest reliability. Nasreddine et al. (2005) reported an intraclass correlation coefficient (ICC) of 0.92 in their original validation study. Further, the MoCA was highly correlated with the MMSE (*r* = 0.83).

#### COVID-19 survey

The COVID-19 experience survey with 32 questions was conducted to evaluate demographic features, COVID-19 coping behaviors, vaccination status, and opinions related to the pandemic. The survey was designed through a comprehensive literature review which involved searching PubMed, EMBASE, and PsycINFO databases using the following keywords and their combinations: (“COVID-19” OR “SARS-CoV-2” OR “coronavirus” OR “pandemic”) AND (“mental health” OR “psychological health” OR “psychological impact” OR “anxiety” OR “depression” OR “stress, psychological” OR “Stress Disorders, Post-Traumatic” OR “burnout, psychological” OR “burnout, professional” OR “adaptation, psychological” OR “coping strategies” OR “resilience” OR “health promotion” OR “social isolation” OR “quarantine”).

We identified several factors related to SARS-COV-2 infection, regulations, and corollaries that impacted mental health during the COVID-19 pandemic: (i) work as an essential worker (EW) during the lockdown, (McCormack et al., 2020), (ii) experiencing unemployment or income loss, (iii) testing positive for SARS-CoV-2, and (iv) engaging in wellness activities to promote well-being (Giuntella et al., 2021). In the present study, the influence of four potential contributing variables for mental health outcomes was investigated.

### Procedures

Patients enrolled in the WD registry undergo annual comprehensive evaluations, including medical assessment, neurological examination, and psychometric measures. Although all assessments were initially performed in person, due to the COVID-19 pandemic, participants were offered an additional remote assessment option beginning in March 2020 to ensure the study’s continuation. The COVID-19 experience survey was distributed via the Qualtrics platform (Qualtrics International Inc., co-headquarters in Provo, Utah, Seattle, Washington) to all adult patients enrolled in the WD registry at YNHH as of November 2022.

### Statistical analyses

The analyses tested the time effect on participants’ mental state by comparing the three repeated measure scores for each scale. Furthermore, participants were separated into two groups across four categories, considering their status as EWs or non-essential workers (non-EWs), the presence or absence of income loss, engagement in wellness activities (e.g., exercise, gardening, outdoor activities, fasting, a healthy diet, meditation, yoga, and spiritual activities), and whether they contracted SARS-CoV-2 during the pandemic. The grouping variable was between subjects. A mixed 3 × 2 ANOVA was performed, comparing three time periods [First Before COVID-19 pandemic (BC), Second BC, and After COVID-19 (AC)] and two groups, separately for each dependent variable. The groups varied depending on the analysis.

Before reporting the findings, we would like to highlight the possible concerns related to crosstabulation and the byproduct of the groups. We meticulously examined the crosstabulation and conducted chi-square analyses for all four categorical grouping variables in a 2 × 2 analysis. No significant overlap was observed among the grouping variables. The relationship between being an essential worker and being infected by COVID-19 exhibited the highest overlap, with 80% of essential workers and 60% of non-essential workers being infected. However, this overlap was not statistically significant, as the majority of individuals in both groups (workers and non-workers) were infected.

## Results

We divided our analysis into two sections: first, we report findings of the whole cohort (*N* = 62) who responded to our survey on knowledge, attitudes, and perceptions of WD patients towards the COVID-19 pandemic and vaccinations. The second part of our analysis reports on a sub-cohort (*N* = 33) that had multiple data points before and after the onset of the pandemic. This group was examined to evaluate the mental impacts of the COVID-19 pandemic.

### Wilson’s Disease patients’ views on COVID-19 pandemic and vaccinations

#### Patient experiences through the COVID-19 pandemic

The descriptive statistics of the participants’ COVID-19 experience are summarized in Table 2. One-third of the study participants were considered EWs during the pandemic (Essential healthcare workers: 14%, Essential non-healthcare workers: 22%). A total of 19% of the participants experienced job loss and overall, 31% experienced some level of income loss during the pandemic. Most of the participants continued living at the same location (82%) and shared their homes with someone else (84%). Over half of the participants were infected with SARS-CoV-2 (61%) or experienced an infection in their household (61%), and only one patient was hospitalized for COVID-19. Five percent of the participants lost family members during the pandemic. Most of the participants’ WD treatment stayed the same (81%). Approximately half of the study participants engaged in wellness activities (42%) or adopted a pet (24%) (Table 2).

**Table 2:** The summary of COVID-19 experience and vaccination history of the sample.

		*N* (62)	%
Essential healthcare worker	No	53	85.50%
	Yes	9	14.50%
Essential nonhealthcare worker	No	48	77.40%
	Yes	14	22.60%
Job loss	No	50	80.60%
	Yes	12	19.40%
Income loss	No	40	64.50%
	Yes	19	30.60%
	Prefer not to say	3	4.80%
Relocation	No	51	82.30%
	Yes/permanent	8	12.90%
	Yes/temporary	3	4.80%
Living during the pandemic	Living alone	10	16.10%
	With someone else	52	83.90%
WD treatment change	No	50	80.60%
	Yes	12	19.40%
SARS-CoV-2 infection	No	24	38.70%
	Yes	38	61.30%
SARS-CoV-2 within the household	No	24	38.70%
	Yes	38	61.30%
Family loss	No	59	95.20%
	Yes	3	4.80%
Hospitalized	No	37	59.70%
	Yes	1	1.60%
	Missing	24	38.70%
Pet adoption	No	47	75.80%
	Yes	15	24.20%
Wellness activity	No	36	58.10%
	Yes	26	41.90%

#### Vaccination attitudes and perceptions among patients

Almost all the patients were vaccinated for SARS-CoV-2 (94%). The most common vaccination types were Pfizer and Moderna. Most participants considered vaccination safe (77%); however, 18% of participants were not sure yet, and 5% thought that vaccination was unsafe. Most of the participants stated that vaccination was necessary (69%), while some patients considered it unnecessary (7%) or were not sure about it (15%) (See Table 3).

**Table 3:** The summary of vaccination history of the sample.

Vaccination	No	4	6.50%
	Yes	58	93.50%
Dose 1	No Vac	5	8.10%
	J&J	6	9.70%
	Moderna	19	30.60%
	Pfizer	32	51.60%
Dose 2	No vac	6	9.70%
	Moderna	26	41.90%
	Pfizer	30	48.40%
Dose 3	No vac	12	19.40%
	Moderna	23	37.10%
	Pfizer	27	43.50%
Dose 4	No vac	39	62.90%
	0vavax	1	1.60%
	Moderna	9	14.50%
	Pfizer	13	21.00%
Dose 5	No Vac	54	87.10%
	Moderna	3	4.80%
	Pfizer	5	8.10%
COVID-19 opinion for own health	Not serious	19	30.60%
	Serious	17	27.40%
	Varies on a case-by-case base	26	41.90%
COVID-19 opinion for others health	Not serious	2	3.20%
	Serious	19	30.60%
	Varies on a case-by-case base	41	66.10%
Vaccination safety	No	3	4.80%
	Yes	48	77.40%
	Unsure	11	17.70%
Vaccination necessity	I am unsure	9	14.50%
	Other	6	9.70%
	Vaccination is necessary	43	69.40%
	Vaccination is unnecessary	4	6.50%

#### Patient perceptions of SARS-CoV-2 infection

Patients’ opinions on contracting SARS-CoV-2 varied as well. A total of 31% of the participants considered it not serious for their own health, while only 3% believed it was not serious for others. A total of 27% of the participants concurred that the virus posed a significant threat to their own health, and 31% stated that was serious for others. A total of 42% of the patients noted that risk associated with the virus varied case by case for their own health, and 66% stated the same for others’ health (see Table 3).

### The impact of COVID-19 on mental health

#### Essential workers in the COVID-19 pandemic

**Figure 2:**
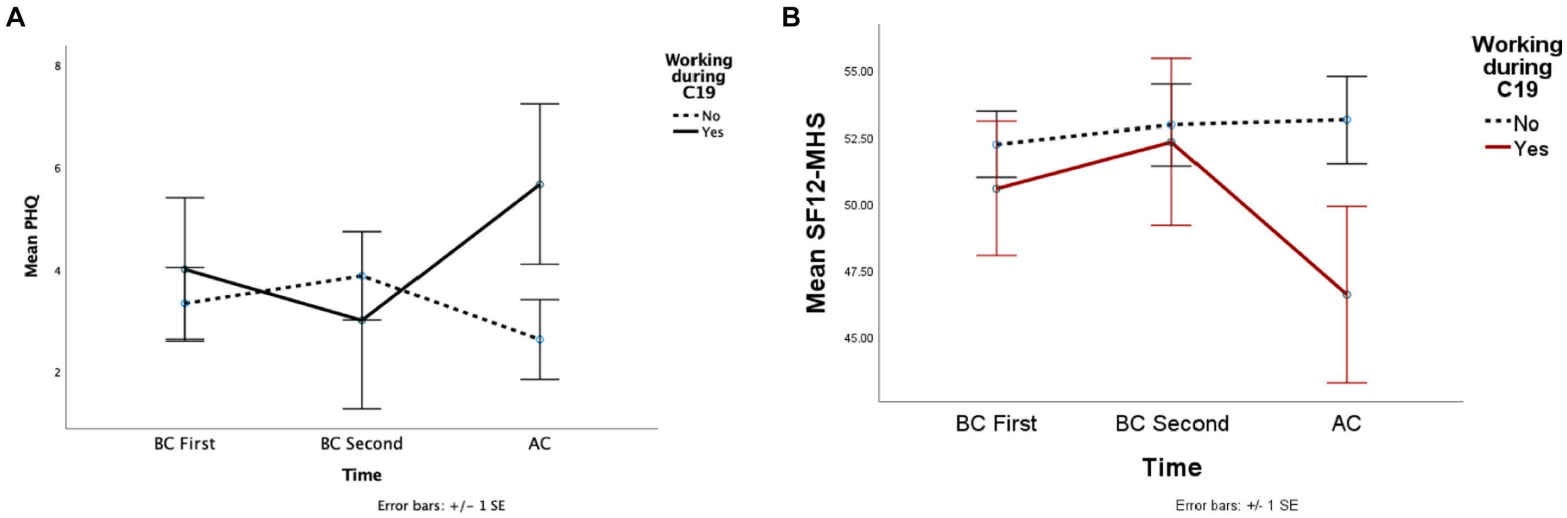
This figure depicted the mean depression **(A)** and perceived mental health **(B)** scores across the groups whether working during the pandemic.

Overall, 10 of the participants had to work during the pandemic, and 22 did not have to work. Results from the working condition analysis indicated a significant interaction effect on depression levels (*F* (2, 56) = 5.9, *p* = 0.005, *η_p_^2^* = 0.17). The two measures BC were similar for EW, and non-EW groups with a minimum mean difference (*MD*); however, participants who had to work AC revealed higher depression score than those who did not work (*MD* = 1.82) (Table 4). Furthermore, the AC depression score of the working participants was significantly higher than the second BC (*MD* = 2.67) (Figure 2A).

**Table 4:** The summary of psychiatric measures descriptive statistics across COVID-19 experience groups.

	Time					
	BC first		BC Second		AC	
	No	Yes	No	Yes	No	Yes
Essential worker						
MOCA	27 (2.32)	27.5 (1.05)	26.96 (2.32)	27.67 (1.37)	27.17 (1.75)	27.33 (1.03)
PHQ9	3.33 (3.16)	4 (4.56)	3.88 (4.43)	3 (3.35)	2.63 (3.16)	5.67 (6.02)
PSS	10.96 (6.83)	10.5 (10.67)	11.04 (6.67)	10.83 (8.75)	10.24 (5.39)	11.17 (11.27)
GAD7	3.42 (3.67)	3.83 (6.14)	3.38 (4.46)	3.17 (5.19)	2.63 (3.03)	4.83 (6.18)
SF12-PHS	53.26 (9.22)	51.46 (6.42)	53.33 (9.66)	53.21 (4.06)	53.35 (8.64)	51.83 (7.78)
SF12-MHS	52.21 (5.62)	50.56 (8.19)	52.94 (7.69)	52.3 (7.36)	53.12 (7.02)	46.58 (12.1)
Income loss						
MOCA	26.95 (2.15)	27.4 (1.12)	27 (2.31)	27.3 (1.95)	27.21 (1.81)	27.2 (1.23)
PHQ9	3.10 (2.5)	4.2 (6.1)	3.15 (3.3)	4.8 (5.63)	2.2 (1.91)	5.3 (5.98)
PSS	9.38 (6.18)	14 (9.33)	9.24 (6.24)	14.7 (7.24)	8.62 (4.9)	14.2 (8.47)
GAD7	2.35 (2.3)	5.8 (5.96)	2.2 (2.76)	5.6 (6.41)	1.85 (1.6)	5.5 (5.66)
SF12-PHS	54.44 (7.4)	49.71 (10.61)	54.76 (7.8)	50.26 (10.36)	54.71 (7.56)	49.58 (9.34)
SF12-MHS	53.13 (5.73)	49.31 (6.25)	54.17 (7.67)	49.97 (6.64)	54.19 (5.81)	46.95 (11)
Wellness activity initiation						
MOCA	27.31 (1.96)	26.85 (2.34)	27.31 (2.57)	26.85 (1.57)	27.13 (1.36)	27.31 (1.93)
PHQ9	3.65 (3.62)	3.23 (3.22)	3.82 (5.22)	3.54 (2.47)	3.35 (3.52)	3.08 (4.63)
PSS	11.67 (7.32)	9.77 (7.93)	10.33 (7.62)	11.92 (6.09)	10.5 (6.16)	10.31 (7.6)
GAD7	3.47 (4.11)	3.54 (4.37)	3.47 (5.2)	3.15 (3.65)	2.35 (2.71)	4 (4.9)
SF12-PHS	51.59 (10.43)	54.75 (5.29)	51.76 (10.57)	55.45 (5.16)	52.08 (8.2)	54.41 (8.76)
SF12-MHS	51.82 (6)	51.99 (6.43)	54 (8.15)	51.17 (6.48)	52.51 (7.89)	50.94 (9.33)
SARS-CoV-2 infection						
MOCA	26 (2.5)	27.6 (1.76)	25.67 (2.6)	27.75 (1.6)	27.11 (1.54)	27.25 (1.68)
PHQ9	3 (2.16)	3.7 (3.91)	3.2 (1.93)	3.95 (4.99)	3 (1.63)	3.35 (4.76)
PSS	11.3 (6.75)	10.67 (8)	10.6 (5.87)	11.19 (7.55)	9.2 (5.59)	11 (7.19)
GAD7	3.3 (3.02)	3.6 (4.68)	2.6 (2.84)	3.7 (5.19)	2.2 (1.93)	3.5 (4.48)
SF12-PHS	54.54 (4.53)	52.14 (10.09)	54.31 (6.53)	52.83 (9.81)	51.92 (8.18)	53.6 (8.61)
SF12-MHS	51.13 (7.19)	52.26 (5.63)	52.91 (6.03)	52.77 (8.26)	52.37 (6.66)	51.61 (9.27)

The interaction of participants’ mental health perception scores approached the significance level (*F* (2, 58) = 2.77, *p* = 0.07, *η_p_^2^* = 0.09). Before COVID-19, the scores were similar between the two groups, and mean differences were minimal. However, EW’s mental health scores decreased AC compared to the second BC score (*MD* = 5.72). After COVID-19, there was a considerable difference between the scores of EW and non-EW groups (*MD* = 4.94), although the difference was not significant. The other dependent variables did not reveal significant main effects or interactions (Figure 2B).

#### The impact of income loss on mental health during the pandemic

**Figure 3:**
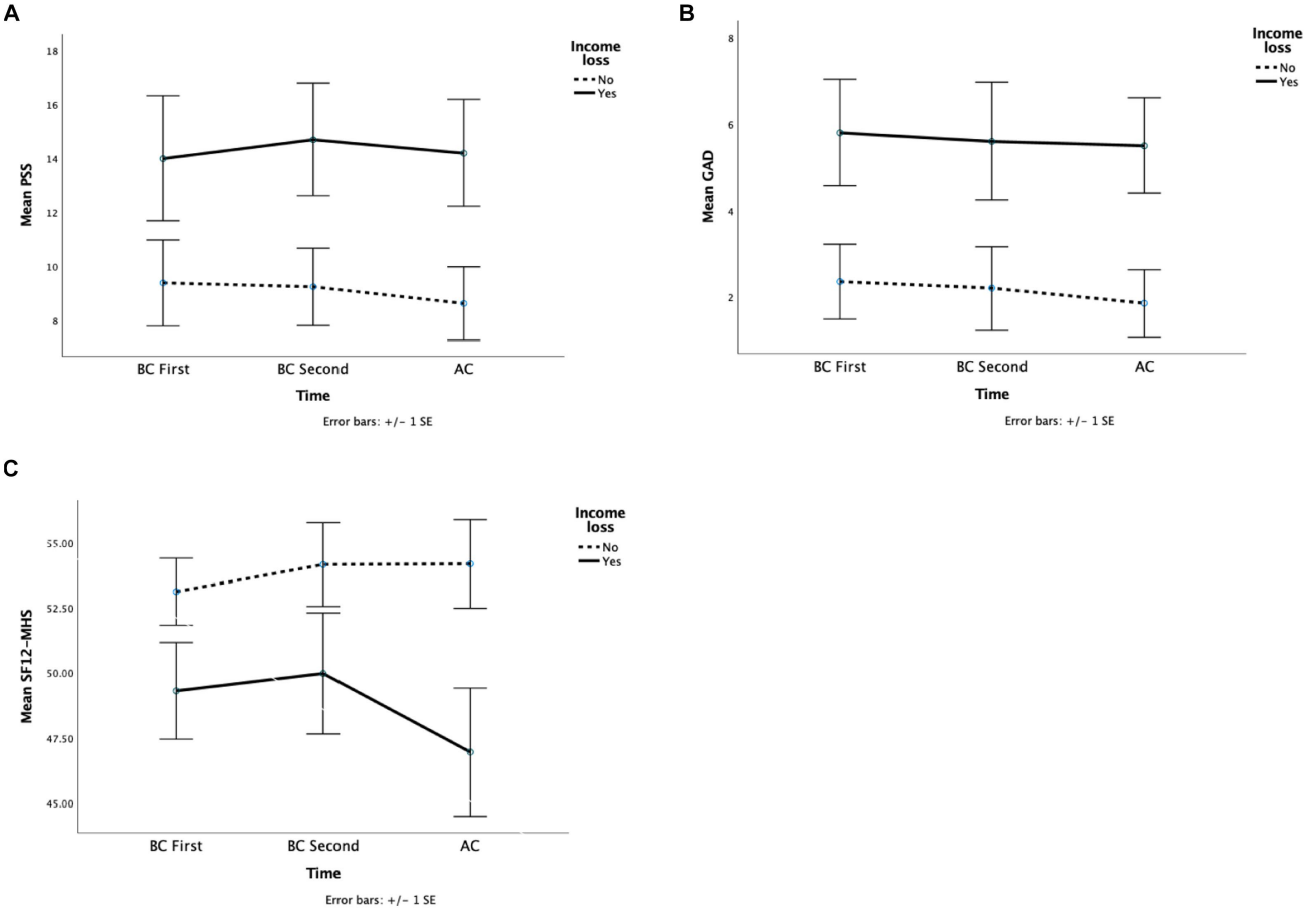
This figure depicted the mean stress **(A)**, anxiety **(B)**, and perceived mental health **(C)** scores across the groups whether lost jobs during the pandemic.

Overall, 11 participants experienced some sort of income loss during the pandemic, and 21 did not experience it (Table 4). An income loss main effect was observed on various dependent variables. Patients who experienced an income loss scored higher in stress (Figure 3A) (*F* (1, 29) = 5.59, *p* = 0.02, *η_p_^2^* = 0.16, *MD* = 5.39), anxiety (Figure 3B) (*F* (1, 28) = 6.62, *p* = 0.02, *η_p_^2^* = 0.19, *MD* = 3.51) and mental health perception (Figure 3C) (*F* (1, 29) = 4.63, *p* = 0.04, *η_p_^2^* = 0.14, *MD* = 5.06) tests than those who did not (Table 5). The other dependent variables did not reveal significant main effects or interactions.

**Table 5:** The summary of psychiatric measures ANOVA results across COVID-19 experience groups.

	Time effect	Group main effect	Interaction
Essential worker			
MOCA	*F* (2, 54) = 0.01, *p* = 1, η*_p_^2^* = 0	*F* (1, 27) = 0.48, *p* = 0.49, *η_p_^2^* = 0.02	*F* (2, 54) = 0.13, *p* = 0.88, *η_p_^2^* = 0.01
PHQ9	*F* (2, 56) = 0.79, *p* = 0.46, η*_p_^2^* = 0.03	*F* (1, 28) = 0.34, *p* = 0.57, *η_p_^2^* = 0.01	*F* (2, 56) = 5.9, *p* = 0.005, *η_p_^2^* = 0.17
PSS	*F* (2, 58) = 0.02, *p* = 0.98, *η_p_^2^* = 0	*F* (1, 29) = 0, *p* = 0.98, *η_p_^2^* = 0	*F* (2, 58) = 0.15, *p* = 0.86, *η_p_^2^* = 0.01
GAD7	*F* (2, 56) = 0.28, *p* = 0.76, *η_p_^2^* = 0.01	*F* (1, 28) = 0.21, *p* = 0.65, *η_p_^2^* = 0.01	*F* (2, 56) = 1.91, *p* = 0.16, *η_p_^2^* = 0.06
SF12-PHS	*F* (2, 58) = 0.27, *p* = 0.77, *η_p_^2^* = 0.01	*F* (1, 29) = 0.1, *p* = 0.76, *η_p_^2^* = 0	*F* (2, 58) = 0.24, *p* = 0.79, *η_p_^2^* = 0.01
SF12-MHS	*F* (2, 58) = 2.14, *p* = 0.13, *η_p_^2^* = 0.07	*F* (1, 29) = 0.99, *p* = 0.33, *η_p_^2^* = 0.03	*F* (2, 58) = 2.77, *p* = 0.07, *η_p_^2^* = 0.09
Income loss			
MOCA	*F* (2, 54) = 0.01, *p* = 0.99, *η_p_^2^* = 0.005	*F* (1, 27) = 0.19, *p* = 0.66, *η_p_^2^* = 0.01	*F* (2, 54) = 0.12, *p* = 0.88, *η_p_^2^* = 0.01
PHQ9	*F* (2, 56) = 0.21, *p* = 0.81, *η_p_^2^* = 0.01	*F* (1, 28) = 2.11, *p* = 0.16, *η_p_^2^* = 0.07	*F* (2, 56) = 1.99, *p* = 0.15, *η_p_^2^* = 0.07
PSS	*F* (2, 58) = 0.12, *p* = 0.89, *η_p_^2^* = 0	*F* (1, 29) = 5.59, *p* = 0.02, *η_p_^2^* = 0.16	*F* (2, 58) = 0.1, *p* = 0.9, *η_p_^2^* = 0
GAD7	*F* (2, 56) = 0.25, *p* = 0.78, *η_p_^2^* = 0.01	*F* (1, 28) = 6.62, *p* = 0.02, *η_p_^2^* = 0.19	*F* (2, 56) = 0.03, *p* = 0.97, *η_p_^2^* = 0
SF12-PHS	*F* (2, 58) = 0.09, *p* = 0.91, *η_p_^2^* = 0	*F* (1, 29) = 2.56, *p* = 0.12, *η_p_^2^* = 0.08	*F* (2, 58) = 0.04, *p* = 0.96, *η_p_^2^* = 0
SF12-MHS	*F* (2, 58) = 0.84, *p* = 0.44, *η_p_^2^* = 0.03	*F* (1, 29) = 4.63, *p* = 0.04, *η_p_^2^* = 0.14	*F* (2, 58) = 1.31, *p* = 0.28, *η_p_^2^* = 0.04
Wellness activity initiation			
MOCA	*F* (2, 54) = 0.06, *p* = 0.94, *η_p_^2^* = 0	*F* (1, 27) = 0.21, *p* = 0.65, *η_p_^2^* = 0.01	*F* (2, 54) = 0.35, *p* = 0.71, *η_p_^2^* = 0.01
PHQ9	*F* (2, 56) = 0.42, *p* = 0.66, *η_p_^2^* = 0.01	*F* (1, 28) = 0.06, *p* = 0.81, *η_p_^2^* = 0	*F* (2, 56) = 0.01, *p* = 0.99, *η_p_^2^* = 0
PSS	*F* (2, 58) = 0.23, *p* = 0.8, *η_p_^2^* = 0.01	*F* (1, 29) = 0.01, *p* = 0.94, *η_p_^2^* = 0	*F* (2, 58) = 1.32, *p* = 0.27, *η_p_^2^* = 0.04
GAD7	*F* (2, 56) = 0.2, *p* = 0.82, *η_p_^2^* = 0.01	*F* (1, 28) = 0.11, *p* = 0.75, *η_p_^2^* = 0	*F* (2, 56) = 2.02, *p* = 0.14, *η_p_^2^* = 0.07
SF12-PHS	*F* (2, 58) = 0.1, *p* = 0.9, *η_p_^2^* = 0	*F* (1, 29) = 1.11, *p* = 0.3, *η_p_^2^* = 0.04	*F* (2, 58) = 0.22, *p* = 0.81, *η_p_^2^* = 0.01
SF12-MHS	*F* (2, 58) = 0.33, *p* = 0.72, *η_p_^2^* = 0.01	*F* (1, 29) = 0.35, *p* = 0.56, *η_p_^2^* = 0.01	*F* (2, 58) = 0.91, *p* = 0.41, *η_p_^2^* = 0.03
SARS-CoV-2 infection			
MOCA	*F* (2, 54) = 0.58, *p* = 0.56, *η_p_^2^* = 0.02	*F* (1, 27) = 5.82, *p* = 0.02, *η_p_^2^* = 0.18	*F* (2, 54) = 2.37, *p* = 0.1, *η_p_^2^* = 0.08
PHQ9	*F* (2, 56) = 0.28, *p* = 0.76, *η_p_^2^* = 0.01	*F* (1, 28) = 0.19, *p* = 0.67, *η_p_^2^* = 0.01	*F* (2, 56) = 0.08, *p* = 0.92, *η_p_^2^* = 0
PSS	*F* (2, 58) = 0.36, *p* = 0.7, *η_p_^2^* = 0.01	*F* (1, 29) = 0.06, *p* = 0.81, *η_p_^2^* = 0	*F* (2, 58) = 0.56, *p* = 0.57, *η_p_^2^* = 0.02
GAD7	*F* (2, 56) = 0.58, *p* = 0.57, *η_p_^2^* = 0.02	*F* (1, 28) = 0.36, *p* = 0.55, *η_p_^2^* = 0.01	*F* (2, 56) = 0.45, *p* = 0.64, *η_p_^2^* = 0.02
SF12-PHS	*F* (2, 58) = 0.31, *p* = 0.73, *η_p_^2^* = 0.01	*F* (1, 29) = 0.06, *p* = 0.81, *η_p_^2^* = 0	*F* (2, 58) = 2.02, *p* = 0.14, *η_p_^2^* = 0.07
SF12-MHS	*F* (2, 58) = 0.51, *p* = 0.6, *η_p_^2^* = 0.02	*F* (1, 29) = 0, *p* = 0.98, *η_p_^2^* = 0	*F* (2, 58) = 0.33, *p* = 0.72, *η_p_^2^* = 0.01

#### Initiating wellness activities (health promoting activities during the pandemic)

**Figure 4:**
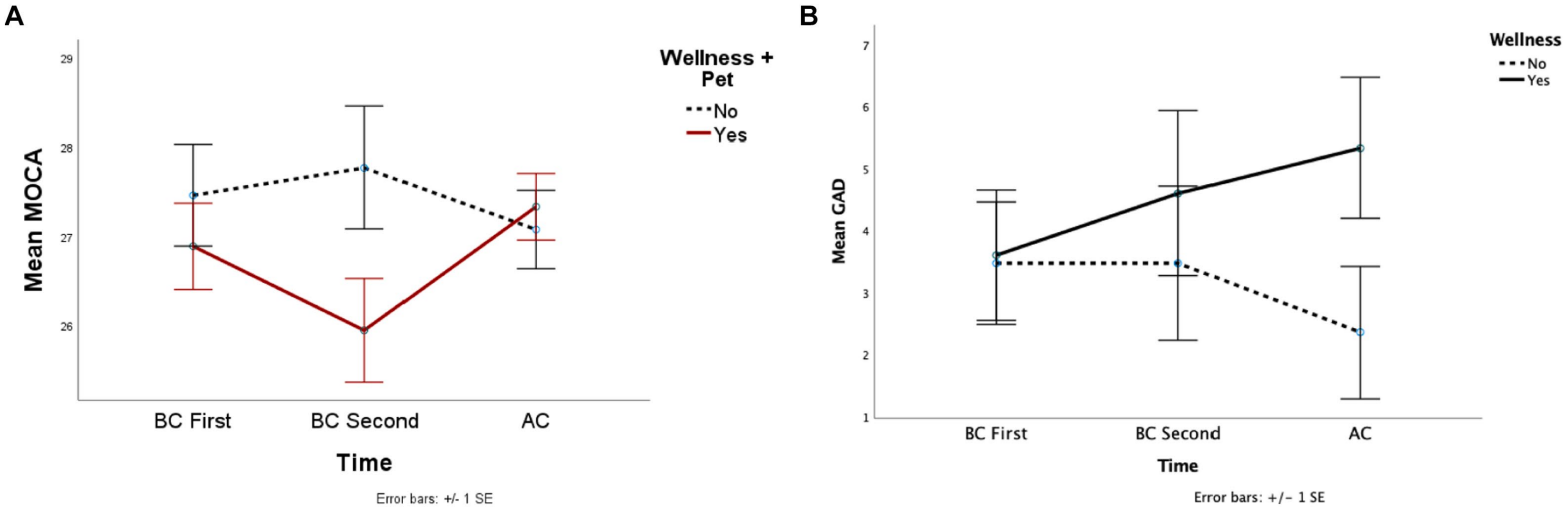
This figure depicted the mean cognitive screening **(A)**, and anxiety **(B)** scores across the groups whether initiate a wellness activity during the pandemic.

15 participants initiated at least one type of wellness activity during the pandemic, while 17 did not. The activities included exercise, gardening, outdoor activities, fasting, a healthy diet, meditation, yoga, and spiritual activities. The interaction of initiating a wellness activity and time approached a significance level in anxiety (*F* (2, 60) = 2.96, *p* = 0.06, *η_p_^2^* = 0.09) and cognitive screening scores (*F* (2, 58) = 2.45, *p* = 0.09, *η_p_^2^* = 0.08). Participants who initiated a wellness activity during the COVID-19 pandemic displayed higher anxiety scores. Compared to their pre-pandemic states (*MD* = 1.73) (Table 4). Further, those participants were more anxious than those who did not start a wellness activity after COVID-19 (*MD* = 2.98) (Figure 4B). After COVID (AC) cognitive screening score of the participants who initiated a wellness activity was significantly higher than their second BC score (*MD* = 1.39). However, their cognitive screening scores did not reveal a significant difference from those who did not start a wellness activity (Figure 4A). The other dependent variables did not reveal significant main effects or interactions.

#### Impact of SARS-CoV-2 infection on mental health outcomes

**Figure 5:**
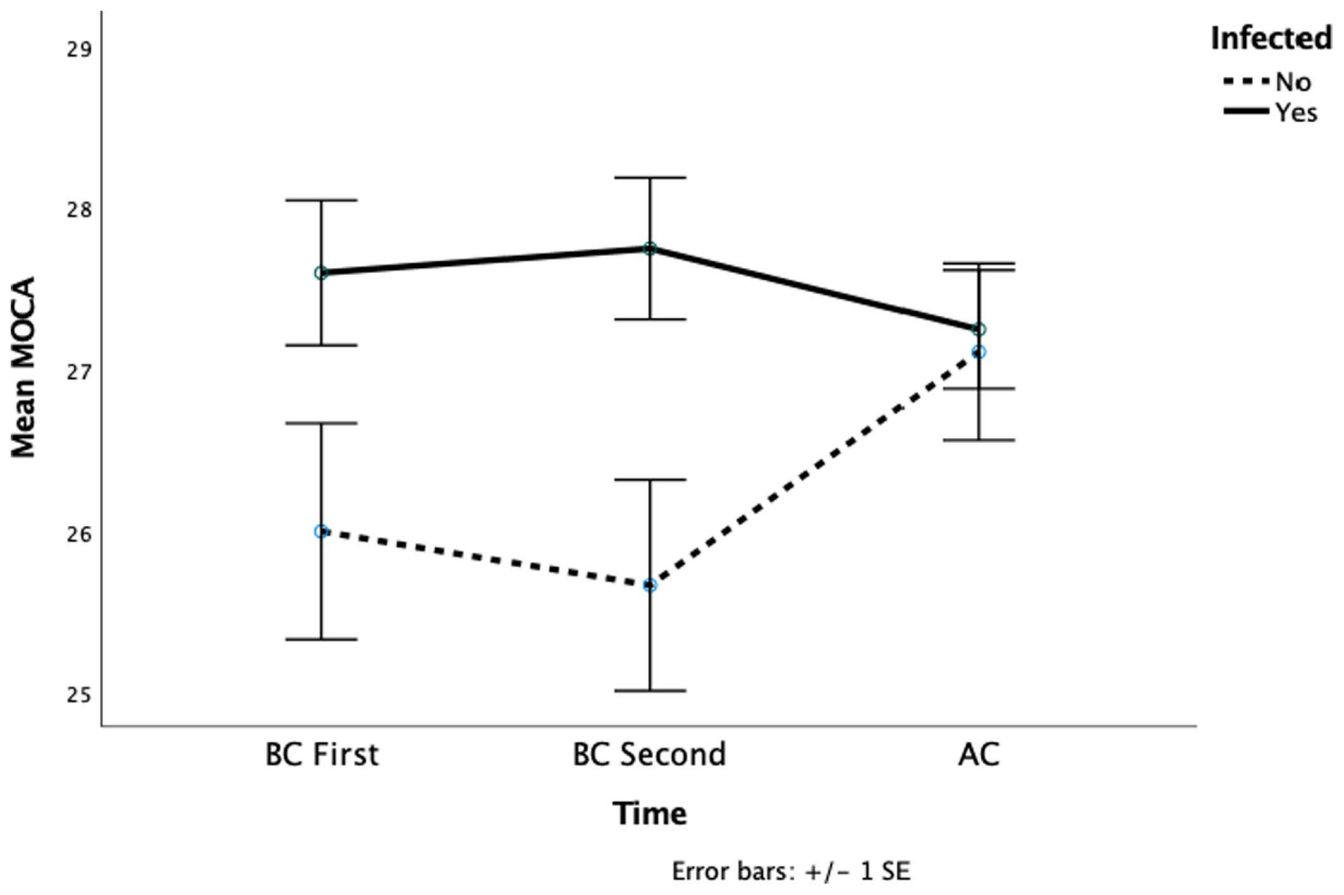
This figure depicted the mean cognitive screening across the groups whether infected by COVID-19.

Out of 33 participants, 21 have a positive SARS-CoV-2 test result. Infection status revealed a main effect on cognitive screening (*F* (1, 27) = 5.82, *p* = 0.02, *η_p_^2^* = 0.18). The interaction approached significance (*F* (2, 54) = 2.37, *p* = 0.09, *η_p_^2^* = 0.08) (Table 5). Patients who were infected scored higher on the cognitive screening scale before the COVID-19 pandemic than those who did not (*MD* = 1.44) (Table 3). This difference disappeared in AC scores, where uninfected patients’ scores increased (*MD* = 2.36) while infected patients’ scores decreased slightly (*MD* = 0.5) (Figure 5). The other dependent variables did not reveal significant main effects or interactions.

## Discussion

To our knowledge, this study represents the first attempt to investigate the vaccination attitudes, experiences during the COVID-19 pandemic, and perceptions among individuals with Wilson’s disease (WD), alongside examining the neuropsychiatric effects of the pandemic on this particular population. Our findings unveiled a remarkably high vaccination rate among WD patients, with 94% of participants reporting being vaccinated. Furthermore, a substantial proportion of participants expressed confidence in the safety (77%) and necessity (69%) of COVID-19 vaccination. The pandemic appeared to have adverse effects on the mental well-being of WD patients, as evidenced by increased rates of depression and lower self-perceived mental health. Additionally, a subset of participants reported experiencing elevated levels of stress and anxiety, prompting them to adopt various wellness activities to cope with these challenges. Moreover, our study identified some variations in cognitive function among WD patients.

It’s worth noting that our patient population differed in several respects from the general population. Predominantly comprised of individuals of Caucasian ethnicity (87%) (Beinhardt et al., 2014), this population also exhibited higher levels of education (Census Bureau Releases New Educational Attainment Data, n.d.) and greater income compared to national averages (Income in the United States: 2021, n.d.).

A significant portion of the U.S. workforce, totaling approximately 55 million individuals (39% of the workforce), was classified as essential during the pandemic, continuing to work outside their homes despite the risks (Who are essential workers? A comprehensive look at their wages, demographics, and unionization rates|Economic Policy Institute, n.d.). This essential workforce included healthcare professionals, first responders, grocery store staff, public transit operators, and others vital for maintaining critical services and infrastructure. Within our patient cohort, 36% were classified as essential workers, providing a unique opportunity to examine the pandemic’s neuropsychiatric impact on this subgroup.

Recent research indicates that 44% of the U.S. population experienced a decline in household income during the pandemic. Certain demographic groups, including Hispanics, ethnic minorities, unmarried individuals, and those with lower education levels, were disproportionately affected (Bruce et al., 2022). In our study, approximately one-third of participants (31%) reported a decrease in income. Notably, our patient population, predominantly consisting of white, non-Hispanic, married, and highly educated individuals, may have experienced a lesser financial impact compared to the broader population.

Social support has been identified as a protective factor against negative pandemic-related outcomes (Xu and Zhang, 2022), with cohabitation associated with higher levels of life satisfaction (Geprägs et al., 2022). A national survey revealed that 36% of respondents reported significant loneliness during the pandemic (Weissbourd et al., 2021). Within our study group, 84% lived with others during the pandemic. However, due to the small sample size and data limitations, we were unable to analyze the impact of loneliness on mental health and quality of life perceptions.

As of February 2023, it is estimated that 43.9% of the global population has experienced at least one COVID-19 infection (COVID-19 Cumulative Infection Collaborators, 2022). While the relationship between chronic liver disease (CLD) and SARS-CoV-2 risk has yet to be fully established, evidence suggests that (CLD) worsens COVID-19 outcomes, increasing severity and mortality. To our knowledge, there is no information on the prevalence of COVID-19 infection in WD patients. In our cohort, we found the infection risk appears to be slightly higher (61%) than the general population, only one patient in our cohort required hospitalization. Out cohort had a high immunization rate (94%), which surpasses the global vaccination average of 69.4%, and a low rate of COVID-related hospitalization, which supports that vaccination might have provided some level of protection against severe COVID-19 infection (Coronavirus (COVID-19) Vaccinations - Our World in Data, n.d.).

The control of the COVID-19 pandemic through vaccination requires not only the vaccine’s safety and efficacy but also the acceptance of the population. In this study group, just a small percentage of individuals consider vaccination unsafe (5%) or unnecessary (7%). Regular health monitoring, well-established and reliable provider-patient relationships, and high health awareness might have played a significant role in this rate which emphasizes the importance of collaboration between governments, health policymakers, and media to build trust in COVID-19 vaccination by spreading accurate messages through trusted channels (Sallam, 2021).

Our study also informs about patient’s perceptions of the COVID-19 Pandemic. Current findings suggest that having a chronic medical condition or poor health perception was linked to higher risk awareness (Cipolletta et al., 2022). Another plausible interpretation for the heightened risk awareness observed in our sample could be attributed to their higher education levels. The individuals being investigated in this study tend to have a relatively higher level of education compared to the broader population. This elevated educational status might enhance their awareness of the severe impact of the virus, prompting them to take preventive measures, such as vaccination. Additionally, this patient population had been followed annually for the registry study and had access to ongoing medical care. Having an established channel of primary care might be our strongest defense against future pandemics. In addition, educating patients on vaccination and early interventions through established primary care might increase individuals’ awareness of ongoing and future pandemics.

Several studies have highlighted a negative association between COVID-19 risk perception and well-being. While heightened awareness of COVID-19 risk is often linked with increased fear, anxiety, and stress (Germani et al., 2020; Motta Zanin et al., 2020), it can also lead to the adoption of coping mechanisms and proactive measures to curb the spread of the virus (Krok and Zarzycka, 2020). The evaluation of perception was conducted 2.5 years following the onset of the pandemic, allowing participants to make more informed assessments regarding preventive measures, infection risks, disease progression, mortality rates, and the safety and efficacy of vaccinations.

A small proportion of the study participants perceived COVID-19 as non-threatening, with the majority acknowledging varying degrees of severity depending on individual cases. In the initial phases of the pandemic, uncertainty and stringent preventive measures contributed to heightened levels of fear, stress, and anxiety. However, as the pandemic evolved, perceptions shifted to recognize the gravity of the disease while considering factors such as the health status of affected individuals.

The COVID-19 pandemic presented significant psychological distress for EWs leading to elevated levels of anxiety, depression (Firew et al., 2020; Muller et al., 2020), insomnia (Zhang et al., 2020), and post-traumatic stress (Carmassi et al., 2020). Heavy workloads, shortages, or excessive use of protective equipment, witnessing patient deaths, separation from family, stigmatization, and personal circumstances such as caring for children or supporting infected family members have added to the already mental distress faced by EWs during the pandemic (Tsamakis et al., 2021). Our study showed that EW had higher depression levels and lower self-perceived mental health during the pandemic, but no significant change was found in their anxiety or stress levels. During the COVID-19 pandemic, while the rate of depression was higher among people with chronic illnesses (Wang et al., 2021) individuals with chronic illnesses may have developed efficient coping skills and have experienced less stress and anxiety during the pandemic.

The COVID-19 pandemic has caused widespread unemployment and decreased income, leading to financial difficulties. This had a negative effect on individuals’ well-being and mental health (Borrescio-Higa et al., 2022). Job uncertainty and financial distress caused by the pandemic, as well as debt management challenges (Andelic and Feeney, 2023), are linked to increased anxiety and depression (Wilson et al., 2020). Similarly, our study found that WD patients who suffered income loss reported elevated levels of stress, anxiety, and a decrease in perceived mental health. Interestingly, the differences in stress, anxiety, and mental health between the individuals who lost their jobs and those who did not were apparent before the pandemic started. These findings suggest that individuals’ elevated stress and anxiety might be the cause of losing jobs, more than the effect.

In our study population, we noted a higher level of stress and anxiety among participants who initiated wellness practices following the onset of the COVID-19 pandemic as compared to both their prior state before the pandemic and those who did not adopt such practices. At first, this finding may appear to contradict existing literature; however, a closer examination can deduce that individuals experiencing elevated levels of stress and anxiety might have adopted wellness practices as a coping mechanism. A subsequent study may be required to see a clear picture of the impact of wellness behavior. Cognitive function appeared to be better in patients who engaged in wellness activities which is consistent with the literature which found that short-term aerobic exercise (Miyazaki et al., 2022) or practicing meditation can enhance the cognitive functions of individuals (Hausswirth et al., 2023).

The COVID-19 pandemic caused lifestyle changes such as less physical activity, social isolation, and disrupted sleep patterns, which affected dietary habits and led to a decline in mental health (Caroppo et al., 2021). A study found a correlation between healthier-sustainable food consumption and lower anxiety, depression, and stress symptoms (Zanatta et al., 2022). Moderate-intensity physical activity, including walking, jogging, and house cleaning, may also be beneficial (Marconcin et al., 2022). Additionally, small studies suggest that meditation, yoga, and breathing exercises may help improve mental health (Desai et al., 2021; Upadhyay et al., 2022).

SARS-CoV-2 infection leads to increased neurocognitive decline in patients (Xie et al., 2022). This effect persists even after 6 months which poses the question of whether or when the neuropsychiatric outcomes of it will return to baseline. The outcome is relevant not only for infected patients but also for healthcare providers and for policymakers tasked with establishing strategies to address this delayed sequela (Taquet et al., 2022). Our study group did not reveal this interaction which may be attributed to just a single patient requiring hospitalization and high vaccination rates in the cohort. As such, studies indicate a higher susceptibility to neurocognitive decline among the elderly, those with comorbidities (Evans et al., 2021), and hospitalized patients (Miskowiak et al., 2023).

Our study has several noteworthy limitations. Since WD is a rare genetic disease, the study has a small sample size which raises concerns about the statistical power of the analysis. Second, the results are specific to WD patients limiting the generalizability to other populations. Third, the mental health analysis was based on a limited sample size of 33 patients who had at least two assessments before the pandemic. This can create a risk of selection bias, and the results may not accurately reflect the mental health of entire WD patients. Although the analysis attempted to control for potential confounding factors, such as EW, income loss, SARS-CoV-2 infection, or health-promoting activities; there might be other factors affecting mental health that were not assessed due to the limited sample size. Finally, the study did not investigate the effects of disease severity and timing of infection and their effects on mental health.

Our study provides a starting point for future studies with a larger cohort for monitoring and addressing the mental health consequences arising from the COVID-19 pandemic among patients with chronic diseases such as WD. Additionally, it highlights the need for further investigations into the long-term cognitive effects of COVID-19 infection and mitigation strategies.

## Conclusion

The COVID-19 pandemic has led to significant consequences on a global scale. In addition to efforts to control and eliminate the virus, it is vital to pay attention to mental health during and after the pandemic. The results of this research hold considerable relevance for policymakers, organizations, and healthcare providers in addressing the challenges posed by the COVID-19 pandemic and its influence on the mental well-being of individuals with WD. It also emphasizes the need for monitoring and effective strategies to improve mental health throughout the post-pandemic recovery period.

## Data availability statement

The raw data supporting the conclusions of this article will be made available by the authors, without undue reservation.

## Ethics statement

The studies involving humans were approved by Yale University Institutional Review Board. The studies were conducted in accordance with the local legislation and institutional requirements. The participants provided their written informed consent to participate in this study.

## Author contributions

AC: Conceptualization, Data curation, Investigation, Methodology, Project administration, Resources, Writing – review & editing. AA: Conceptualization, Investigation, Methodology, Writing – original draft. ST: Formal analysis, Methodology, Visualization, Writing – original draft, Writing – review & editing, Conceptualization. UT: Conceptualization, Data curation, Investigation, Writing – review & editing. SR: Data curation, Funding acquisition, Investigation, Resources, Supervision, Writing – review & editing. MS: Conceptualization, Data curation, Funding acquisition, Resources, Supervision, Writing – review & editing. PZ: Funding acquisition, Resources, Supervision, Writing – review & editing.

## Footnotes

^1^An *a priori* power analysis using the G*Power 3.1 computer program (Faul et al., 2009) demonstrated that a total of 28 people would be needed to detect the effects (*f* = 0.25) with 81% power (1 - β) using a repeated measures (*r* = 0.5 among the conditions, nonsphericity correction ε = 1) ANOVA with a repeated factor, within-between interaction design with alpha at 0.05.
