# Supplementary material for: Wilson Disease and the COVID-19 pandemic: exploring patients’ mental health and vaccination attitudes in a longitudinal study
Source: Front Psychol. 2024 May 13;15:1326802. doi: 10.3389/fpsyg.2024.1326802 (PMC11129684; doi:10.3389/fpsyg.2024.1326802)
Supplement: Supplementary file 1 [file Table_1.docx]

Covid-19 Survey

Start of Block: Default Question Block

Q1

Welcome to the research study!  
 
We are interested in understanding the impacts of COVID-19 pandemic on patients with Wilson Disease.  You will be asked to answer some questions on your COVID exposure and perception of disease and vaccination. Please be assured that your responses will be kept completely confidential. The information gathered will be used for current and future Wilson Disease and COVID-19 related research.
  
 The survey should not take  more than 10-15 minutes to complete. Your participation in this research is voluntary. You have the right to withdraw at any point during the survey, for any reason, and without any prejudice. If you would like to discuss this research, please contact Principal **Investigator** Dr. Michael Schilsky at michael.schilsky@yale.edu.
  
 By clicking the button below, you acknowledge that your participation in the study is voluntary, you are 18 years of age or older, and that you are aware that you may choose to terminate your participation in the survey at any time and for any reason.
  
 Please note that this survey will be best displayed on a laptop or desktop computer however you should be also able to complete it on a mobile device. Please reach us at (203) 376-6063 if you encounter any technical difficulty.   

- I consent, begin the study (1)
- I do not consent, I do not wish to participate (2)

| Page Break |  |
| --- | --- |

Q2 WDR Study ID:

________________________________________________________________

Q3 Which race or ethnicity best describes you? ***(Please choose only one)***

- American Indian or Alaskan Native (1)
- Asian / Pacific Islander (2)
- Black or African American (3)
- Hispanic (4)
- White / Caucasian (5)
- Multiple ethnicity / Other (please specify) (6) __________________________________________________
- Prefer not to answer (7)

Q4 What is the highest degree or level of school you have completed? ***(If you’re currently enrolled in school, please indicate the highest degree you have received)***

- Less than a high school diploma (1)
- High school degree or equivalent (e.g. GED) (2)
- Some college, no degree (3)
- Associate degree (e.g. AA, AS) (4)
- Bachelor’s degree (e.g. BA, BS) (5)
- Master’s degree (e.g. MA, MS, MEd) (6)
- Professional degree (e.g. MD, DDS, DVM) (7)
- Doctorate (e.g. PhD, EdD) (8)
- Prefer not to answer (9)

Q5 Marital Status
 ***Please mark your status at both time points.***

|  | On March 2020 (1) | As of Today (2) |
| --- | --- | --- |
| Single (1) |  |  |
| Married, or in a domestic partnership (2) |  |  |
| Separated (3) |  |  |
| Divorced (4) |  |  |
| Widowed (10) |  |  |
| Prefer not to answer (5) |  |  |
| Other (please specify) (6) |  |  |

Q6 Working Status
 ***Please mark your status for both time points.***

|  | On March 2020 (1) | As of Today (2) |
| --- | --- | --- |
| Employed (1) |  |  |
| Unemployed (2) |  |  |
| Student (3) |  |  |
| Retired (4) |  |  |
| Disabled (5) |  |  |
| Unable to work due to other reasons (8) |  |  |
| Prefer not to answer (6) |  |  |
| Other (please specify) (7) |  |  |

Q7 Which of the following best describes your total household income?

- $0 to $19,999 (1)
- $20,000-$49,999 (2)
- $50,000-$89,999 (3)
- $90,000-$129,999 (4)
- $130,000-$149,000 (5)
- $150,000+ (6)
- Prefer not to answer (7)

| Page Break |  |
| --- | --- |

Q8 Did you work as an essential healthcare worker during the pandemic?

- No (1)
- Yes (2)

Q9 Did you work as an essential non-healthcare worker during the pandemic?

- No (1)
- Yes (2)

Q10 Did you experience job loss during pandemic?

- No (1)
- Yes (2)

Q11 Did you experience income loss during pandemic?

- No (3)
- Yes (5)
- Prefer not to answer (4)

Q12 Did you relocate during the pandemic?

- No (1)
- Yes- temporarily (2)
- Yes-permanently (3)

Q13 During the pandemic, with whom did you live?

- By myself (3)
- With someone else (4)

Q14 Did you acquire a pet during the pandemic?

- No (1)
- Yes (2)

Q15 Did you adopt any new wellness practices during the pandemic?

- No (1)
- Yes (2)

| 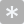 |
| --- |

Q16 Please select all that apply.

- Exercise (1)
- Meditation/Yoga (2)
- Outdoor activities (5)
- Gardening (8)
- Spiritual practices (12)
- Other (please specify) (11) __________________________________________________

| Page Break |  |
| --- | --- |

Q17 During the pandemic, has your Wilson Disease treatment been modified for any reason?

- No (1)
- Yes (2)

| 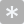 |
| --- |

Q18 How was your treatment modified?

- Physician’s recommendation (1)
- Unable to access healthcare services (2)
- Unable to access medication (3)
- Medication adverse effects (4)
- Change or interruption due to COVID infection. (5)
- Other (please specify) (6) __________________________________________________

| Page Break |  |
| --- | --- |

Q19 Did you receive COVID vaccinations?

- No (21)
- Yes (22)

Q20 Please select the COVID vaccinations you received throughout the pandemic.

|  |  |
| --- | --- |
| Dose 1 (6) | ▼ Moderna (1) ... Novavax (4) |
| Dose 2 (7) | ▼ Moderna (1) ... Novavax (4) |
| Dose 3 (8) | ▼ Moderna (1) ... Novavax (4) |
| Dose 4 (9) | ▼ Moderna (1) ... Novavax (4) |
| Dose 5 (10) | ▼ Moderna (1) ... Novavax (4) |

Q21 Please enter the date as shown. ( ***mm/yyyy***)

- Dose 1 (17) __________________________________________________
- Dose 2 (18) __________________________________________________
- Dose 3 (19) __________________________________________________
- Dose 4 (20) __________________________________________________
- Dose 5 (22) __________________________________________________

| Page Break |  |
| --- | --- |

Q22 Did you get COVID?

- No (1)
- Yes, I tested positive (2)
- Yes, I think I had it but I was not tested (3)

Q23 Please provide the diagnostic test method.

- Hospital based antigen test (2)
- Hospital based PCR test (3)
- Home based antigen test (4)
- I do not know which test I had (5)

Q24 Please enter the date as shown. ***( mm/yyyy ).
 If you have been tested multiple times, insert " , " between the dates.***

- Hospital based antigen test (2) __________________________________________________
- Hospital based PCR test (3) __________________________________________________
- Home based antigen test (4) __________________________________________________
- I do not know which test I had (5) __________________________________________________

Q25 Have you been hospitalized for COVID?

- No (1)
- Yes (2)

Q26 Has anyone in your household had COVID?

- No (1)
- Yes (2)

Q27 Have you ever lost a member of your household or family due to COVID?

- No (1)
- Yes (2)

| Page Break |  |
| --- | --- |

Q28 In your opinion, how serious is COVID-19 infection to your own health?

- Serious (1)
- Not serious (2)
- Varies on a case-by-case basis (3)

Q29 In your opinion, how serious is COVID-19 infection for the health of others?

- Serious (1)
- Not serious (2)
- Varies on a case-by-case basis (3)

Q30 Do you consider the vaccine against COVID-19 to be safe?

- No (1)
- Yes (2)
- I am unsure (3)
- Other(please specify) (4) __________________________________________________

Q31 Do you consider the vaccine against COVID-19 is necessary?

- Vaccination is necessary (1)
- Vaccination is unnecessary (2)
- I am unsure (3)
- Other(please specify) (4) __________________________________________________

Q32 Where do you obtain information regarding COVID-19? ***(check all that apply)***

- Public Health Organizations ( e.g: CDC, local health authorities) (1)
- TV or radio (7)
- Social media (2)
- Healthcare Professionals (3)
- Family members and friends (4)
- Various publicly available websites (5)
- Print media (8)
- Other (please specify) (6) __________________________________________________

Q33 What motivated you to be vaccinated against COVID-19 ?***(check all that apply)***

- To avoid acquiring the COVID infection (1)
- Prevention of hospitalization and severe symptoms due to COVID (2)
- To contribute to eliminating the virus causing COVID (3)
- Desire to be responsible for my community (4)
- To protect my family and friends (5)
- To return to normal conditions (6)
- To be able to travel again (7)
- To be able to go to school or work (8)
- Other (please specify) (9) __________________________________________________

Q34 What is the reason you do not wish to be vaccinated against COVID-19? ***(check all that apply)***

- Do not believe the vaccine to be effective (1)
- Do not believe the vaccine to be safe (2)
- Infected with COVID-19 before and do not need vaccination (3)
- Concerned with adverse effects (4)
- I am motivated to preventative measures (5)
- Immunization ineligible due to allergy, pregnancy, or other conditions (6)
- Not confident in the sufficiency of recent clinical trials to launch the vaccine (7)
- Other (please specify) (8) __________________________________________________

| Page Break |  |
| --- | --- |

Q35
This is the end of the survey. We appreciate your time.

End of Block: Default Question Block
